# Supplementary material for: Are carbon emissions trading and green financial instruments synergistic? -Comprehensive quantitative research based on content analysis
Source: PLoS One. 2024 Mar 7;19(3):e0298601. doi: 10.1371/journal.pone.0298601 (PMC10919601; doi:10.1371/journal.pone.0298601)
Supplement: S1 Data — (ZIP) [file pone.0298601.s001.zip › Data/地级市Z_F工作报告.zip!/║╙─╧/╓ú╓▌╩╨/└┤╫╘╣1⁄2╓┌║┼í╛┬φ┐╦╩2╛▌═°í┐_2003.docx]

各位代表：

　　现在，我代表市人民政府向大会作工作报告，请予审议，并请各位政协委员和列席人员提出意见。

　　一、2002年工作回顾

　　2002年是“十五”计划的第二年，也是我市经济社会发展取得显著成绩的一年。全市人民以邓小平理论和“三个代表”重要思想为指导，认真贯彻党的各项方针政策，着力扩大内需、调整结构、深化改革、扩大开放、优化环境，积极应对入世挑战，国民经济和各项社会事业全面发展，较好地完成了市十一届人大五次会议确定的各项任务。

　　经济持续快速发展，综合实力进一步增强。我们坚持以经济建设为中心，加快发展步伐，全年国内生产总值完成926.8亿元，比上年增长10.9%。其中，第一产业增加值46.2亿元，增长2%；第二产业增加值453.6亿元，增长10.7%；第三产业增加值427亿元，增长12.2%。一二三次产业比重为5.0 : 48.9 : 46.1。城市居民人均可支配收入7772元，增长17.4%；农民人均纯收入3377元，增长7%。“十五”计划顺利推进，国民经济发展势头良好。

　　农业结构进一步调整，农村经济全面发展。农村税费改革顺利推行，农民人均负担减轻34.6%。经济作物和高效农业种植面积增加，促进了农民增收。粮食总产139.9万吨，比上年减产6.7%。全市优质专用小麦播种面积达107.5万亩，增长14.1%。无公害蔬菜基地已发展到8万亩。中牟县被定为全国首批无公害农产品生产基地示范县。西部山区人畜吃水困难基本得到解决。畜牧业发展态势良好，全年肉、蛋、奶总产分别增长5.9%、5.4%和34.7%。人工造林23.3万亩，通道绿化628.4公里。城郊型、都市型农业稳步发展，各类农业龙头企业已发展到110余家。全市乡镇企业完成增加值410.6亿元，增长7.4%;实交税金19.8亿元，增长18%。

　　工业生产快速增长，经济效益明显好转。全部工业增加值378.6亿元，比上年增长10%；其中规模以上工业完成增加值237.9亿元，增长13.5%。工业产品产销衔接良好，产销率逐步提高。经济效益继续回升，规模以上工业实现利润35.2亿元。进一步明确了工业结构调整思路，加快发展高新技术产业，改造、提升传统产业。汽车、铝、食品三大支柱产业逐步发展壮大，增加值占全市规模以上工业的比重为30.2%；金星啤酒荣获国家名牌产品。国家批复高新技术产业化项目10个，高科技项目总投资8.9亿元，争取到国家、省各种政策资金7830万元；安玻飞利浦全平彩显玻壳项目如期点火。企业改革稳步推进。中孚公司、郑州燃气公司先后在沪、港成功上市。企业技术改造和技术进步取得新成效。

　　非公有制经济发展迅速，民营企业不断成长壮大。加强对非公有制经济的指导，加大了对非公有制经济的支持力度。继续在个体、私营企业中实行《收费明白卡》、《缴费登记卡》制度，制止乱收费行为，积极为非公有制经济创造良好的发展环境。全年非公有制经济完成增加值376.5亿元，比上年增长15.2%。思达、金苑面业、三全和思念食品公司等一批民营企业市场竞争中发展壮大。

　　商业经济稳步发展，经营业态不断完善。全市社会消费品零售总额完成430.9亿元，比上年增长11.5%。连锁经营发展迅速，经营业态不断完善，流通现代化水平不断提高。连锁商业的扩张，带动了超市、便利店、专营店等新型业态的快速发展。丹尼斯百货年销售额达11亿元，成为我市首家销售额超10亿元的大型零售商业企业。假日销售屡创新高，招商引资成绩斐然。一批国内外商界巨头相继抢滩郑州，德化商业步行街等新的商业设施建成开放。规范整治商品交易市场，培育龙头市场。城乡市场繁荣，物价稳定，整体服务水平有了明显提升。

　　固定资产投资快速增长，重点项目进展顺利。全社会固定资产投资完成340.7亿元，比上年增长15.2%。基本建设投资119.1亿元，增长17.6%；技术改造投资25.8亿元，增长12.7%；房地产开发投资59.6亿元，增长25.2%。邙山干渠、登封地方铁路、中小学危房改造等一批项目争取到国债资金7.35亿元。重点工程全面推进，完成投资43.9亿元。一批重点基础设施和公益设施建成投产，郑少高速、环城高速等续建项目按计划推进。

　　积极适应入世要求，对外开放水平不断提高。全市直接进出口总值10.4亿美元，增长4.5%；其中直接出口6.9亿美元，增长4.2%。审批外商投资企业67家；实际利用外资1.4亿美元，增长51%。高新技术产业开发区完成国内生产总值31.1亿元，增长34.6%；出口创汇3256万美元，增长30%；财政收入1.6亿元。经济技术开发区完成国内生产总值16.1亿元，增长61%；财政收入1亿元；实际利用外资1882万美元，是上年的4倍。组织参加了省经贸洽谈会、厦洽会、深圳高交会。进一步完善外来投资办事大厅，积极为外商提供服务。对外友好往来不断扩大。顺利通过全国优秀旅游城市复查。嵩山少林景区和黄河游览区荣获国家4A级旅游景区。全年旅游总收入94.5亿元，增长19.2%；旅游外汇收入5400万美元。

　　财政收入快速增长，金融业稳定运行。纺织、煤炭、电力、机械、有色金属、建材、房地产等行业呈快速增长态势，带动了财政收入的稳步增长。全市地方财政收入58.3亿元，为预算的108.3%，按可比口径，比上年增长19%；其中，市本级财政收入27.9亿元，为预算的105.4%，增长17.4%。止2002年底，全市金融机构存款余额1971亿元，比年初增加342亿元；贷款余额1552亿元，比年初增加281亿元。郑州商业银行存款逾80亿元，招商银行郑州分行挂牌营业。

　　城市规划建设管理进一步加强，市容市貌明显改观。坚持城市规划集中管理，首次采用国际方案征集的做法，选定了日本黑川的郑东新区概念规划方案，完成了近期建设规划和起步区详细规划。新区的建设用地报批、出让和征地拆迁安置工作正加紧进行。进入CBD区的18个项目，取得了土地使用权，一些项目已经开工建设。金水东路、黄河东路、CBD内环路首期开工的地段已完成建设任务的80%以上，农业东路、东风东路、市广电中心已开工建设。郑州国际会展中心项目进展顺利，省艺术中心设计方案已基本确定。高校苑区和科技城的前期准备工作正在进行。项目招商和融资工作取得突破性进展，实际到位资金12亿元。坚持新区开发与老区改造并重、协调发展的原则，编制了老城区8个系统专项规划以及大学园区规划。城市基础设施建设进展顺利。环城快速路竣工验收交付使用。中原西路、紫荆山南路、郑花北路等36条道路拓宽改造按期完成。路灯改造、夜景照明工程一期全面完成，基本实现了市区路灯全覆盖。全年新增燃气用户3万户，新增供热面积100万平方米；更新增加公交车辆547台，新辟公交线路25条；复浇道路57万平方米；整修改造人行道板110万平方米。邙山输水干渠南段工程实现供水。东风渠、熊耳河截污工程正加紧施工。全年完成经济适用房31万平方米、廉租住房160套。完成了46条道路行道树的栽植、更新和补植，新增绿地面积242万平方米，被省命名为“省级园林城市”。进一步加强了出租车和户外广告的管理。加大了环境卫生的检查力度和环卫事业的投入。加大对水质、大气污染治理力度，重点流域、重点区域环境质量得到提高，城区环境明显改善。城市日益亮、绿、净、美，市民和来郑客人普遍反映市容市貌变化显著。

　　各项社会事业全面发展，创建文明城市工作取得突破性进展。“两个确保”和城市“低保”得到较好落实，5.33万人实现了就业再就业，市二院被定为低保定点医院。社区“三个一”建设全部完成。人事制度改革进一步推进，市(县)乡机构改革全面完成。科技事业进一步发展，全市科技进步对国民经济增长的贡献率47.1%。全年共组织实施各类科技项目305项，其中国家、省级186项。积极推进教育体制改革，各类教育水平不断提高。完成8.8万平方米的郑州师专新校区、中州大学北校区一期工程建设。市区中小学生饮水问题基本解决，农村初中学校基本配齐了计算机教室，农村中小学教师工资全部实现了由县(市)财政统发。全市人口自然增长率控制在4.03‰以内。文化、卫生、体育等社会事业进一步发展。市杂技馆建成投入使用，市青少年宫主体工程进展顺利。市中医院病房大楼、市一院综合病房楼、市六院病房楼改造等工程已竣工投入使用，市三院门诊楼主体工程竣工。航海体育场竣工并验收为大型优秀工程。在省九运会上，我市取得金牌、奖牌、团体总分第一的好成绩。配备了现代化的警用装备，强化社会治安综合治理，社会大局保持稳定。行政审批制度改革不断深化，新取消审批事项133项。市场经济秩序得到规范，经济发展环境进一步优化。加大反腐败工作力度，坚持不懈地抓好政府廉政建设。城市民兵工作综合性改革顺利完成，民兵预备役建设进一步加强。深入开展“双创”工作，被评为全国创建文明城市工作先进城市。

　　年初向市民承诺办理的十件实事，除教育方面“建设4所初中”，尚有3所正在建设之外，均已完成。其中，全年确保5万人实现就业和再就业，新建经济适用房30万平方米，新增燃气用户2万户，更新公交车辆400台，新辟公交线路20条，新增集中供热面积50万平方米，整修和改造人行道板100万平方米，拓宽改造市区道路35条等已超额完成。

　　新闻出版、广播电视、侨务、对台事务、民族宗教、国家安全、统计、审计、经济协作、社会科学、地方志、档案管理等工作都取得了新的成绩。

　　各位代表：去年政府工作所取得的成绩，是省委、省政府和市委正确领导的结果，是市人大、市政协监督和支持的结果，是全市人民共同努力的结果，是驻郑部队、中央和省驻郑单位、各民主党派、工商联、各人民团体、离退休老同志和海内外各界人士共同努力、鼎力支持的结果。借此机会，我代表市人民政府，向奋斗在全市各条战线的广大工人、农民、知识分子、干部、驻郑人民解放军指战员、武警官兵和公安干警以及社会各界人士致以崇高的敬意，向所有关心、支持我市现代化建设的港、澳特别行政区同胞、台湾同胞、海外侨胞和国际友人表示衷心的感谢！

　　在肯定成绩的同时，我们必须重视经济社会发展中存在的困难和问题：一是农民增收的基础不稳固。去年农民增收主要是农村税费改革、粮食收购实行价内补贴等政策性因素拉动。由于通过提高农产品价格增加农民收入的空间越来越小，如果没有新的增收因素，农民收入持续增长将更加困难。二是国企改革力度不大，效果不理想。中原制药厂破产问题虽已组织专门班子在京做了大量工作，但至今仍未找到出路。三是民间投资尚未完全激活。个体、私营经济发展不够，民间投资增长缓慢，已成为制约全市固定资产投资持续快速增长的关键因素。四是就业和再就业压力较大。随着企业兼并破产力度的逐步加大，全市下岗失业人员将达到7.6万人，其中大龄就业困难人员2.45万人；加之城镇新增劳动力的刚性上升，以及庞大的农村剩余劳动力待转移，就业和再就业形势非常严峻。此外，不少乡镇财政收支矛盾较大，在政府工作中还存在着官僚主义、形式主义作风和以权谋私、铺张浪费等腐败行为。上述问题将对今年政府工作造成不利影响，我们一定要高度重视，在今后的工作中着力予以解决。

　　二、全面建设小康社会及2003年的奋斗目标

　　2003年是全面建设小康社会迈出重要步伐的一年。十六大提出的全面建设小康社会的目标，合潮流，顺民意，极具凝聚力和感召力，必将极大的激发全市干部群众的积极性和创造性。我市作为全国人口第一大省的省会城市，作为经济社会发展具有一定基础的特大城市，面临着建设现代化商贸城和全国区域性中心城市的艰巨任务，经济增长速度理应比全国和全省平均水平更快一些，不仅要使经济更快发展，而且要使民主更加健全、科教更加发达、文化更加繁荣、社会更加和谐、人民生活更加殷实，可持续发展能力不断增强。按照十六大提出的宏伟目标，我市确定的全面建设小康社会目标是：提前实现全面建设小康社会的奋斗目标，国内生产总值提前实现翻两番；到2020年，人均国内生产总值比2000年翻两番；加快工业化、城镇化和商贸城建设，推进农业现代化，在全省率先基本实现工业化。上述预期目标是根据我市经济发展现状及“十五”规划，经过科学预测、认真测算，并经反复论证确定的，惠及全市人民群众，既是积极的，又是可行的，为今后我市的经济社会发展提出了明确要求。只要我们坚持以经济建设为中心，聚精会神搞建设，一心一意谋发展，把发展作为富民兴市的第一要务，就一定能够实现我市全面建设小康社会的目标。

　　根据党的十六大战略部署和我市全面建设小康社会的目标，今年政府工作的指导思想是：高举邓小平理论伟大旗帜，全面贯彻“三个代表”重要思想，认真落实党的十六大精神，按照全面建设小康社会的目标和“十五”计划确定的任务，坚持以结构调整为主线，以机制创新和科技进步为动力，扩大内需，扩大就业，扩大开放，走新型工业化道路，全面繁荣农村经济，加快城镇化进程，进一步优化发展环境，努力实现较高的经济增长速度和较高的经济增长质量，确保社会稳定和生产安全，不断提高人民生活水平，促进社会繁荣进步。

　　今年我市经济社会发展的主要预期目标是：

　　------国内生产总值超千亿元，增长10.5%，综合实力再上一个新台阶；其中第一产业增长3.5%，第二产业增长9.7%，第三产业增长12.1%；

　　------全社会固定资产投资增长15%；

　　------社会消费品零售总额增长11%；

　　------地方财政收入增长11.5%；

　　------外贸出口增长10%，实际利用外资增长15%；

　　------居民消费价格总水平上涨1%左右；

　　------城镇居民人均可支配收入增长8%，农民人均纯收入增长7%；

　　------人口自然增长率控制在6‰以内；

　　------城镇登记失业率控制在3.5%以内，新增就业岗位6万个。

　　实现上述目标，虽然有很多困难和不确定因素，但总体上看，有利条件多于不利条件。党的十六大描绘了全面建设小康社会的宏伟蓝图，增强了全市人民加快发展的信心；国家继续坚持扩大内需的方针，有利于我市进一步扩大投资和消费需求；近年来，我市经济结构调整成效显著，连续实现了较高的经济增长速度，质量和效益明显改善，加快发展的内在活力增强，经济发展的基础更加扎实、稳固；实施东引西进和加入世贸组织有利于我市发挥区位优势，拓展经济发展空间。只要我们趋利避害，应对得当，抓住机遇，扎实工作，就一定能够实现预期目标，为我市全面建设小康社会打下坚实的基础。

　　各位代表：展望新世纪新阶段全面建设小康社会、加快推进社会主义现代化的壮丽前景，我们充满必胜的信心和力量。今年是贯彻落实党的十六大精神的第一年，是实施“十五”计划非常重要和关键的一年，也是本届政府任期的最后一年。做好今年的政府工作，保持经济社会发展的良好势头，对于鼓舞斗志、增强信心，实现全面建设小康社会的重要目标，具有十分重要的意义。我们要在市委领导下，努力做到发展要有新思路，改革要有新突破，开放要有新局面，各项工作要有新举措，迈出我市全面建设小康社会的新步伐。

　　三、2003年的主要工作

　　今年的工作任务十分繁重，全市各级政府要认清形势，抓住机遇，应对挑战，统揽全局，突出重点，着重做好以下几方面工作。

　　(一)加快经济结构调整，促进经济快速发展

　　坚持经济结构调整目标不动摇，加快调整步伐，促使经济结构更加合理，经济质量进一步提高。

　　一是加快工业结构调整步伐，拉长工业短腿。坚持以信息化带动工业化，以工业化促进信息化，用高新技术和先进实用技术改造传统产业，做大做强优势产业和企业，逐步引导我市工业走新型工业化道路。重点抓好中铝河南分公司80万吨氧化铝节能降耗、豫联能源公司10万吨电解铝扩建和郑州铝业公司铝板带箔项目，争取国家10万吨冷轧铝板带项目放在郑州，尽快形成全国铝工业生产基地和科研开发中心。支持宇通公司加快与德国MAN公司合资生产高中档客车专用底盘项目和高档客车生产线建设。支持郑州日产汽车公司加快D22系列新型皮卡的国产化，完成产销计划1.5万台；进而开发多功能皮卡厢式车和商用轿车，完成帕拉丁运动休闲车产销计划1万台。少林汽车公司的中低档客车，要进一步提高质量，扩大规模和市场占有率。抓好以三全、思念为龙头的食品加工业，保持我市速冻食品工业在全国的领先地位。全力支持安飞公司年产250万套的大屏幕玻壳项目，使其做大做强，带动电子信息产业的崛起。加快推进登电集团2×30万KW电厂项目。加大对纺织等传统工业的支持力度，一厂一策，切实帮助困难企业走出困境。要以退内进外为契机，搞好企业技术改造和升级，使企业迈上新台阶，呈现新活力。抓好拖拉机厂、玻璃厂、郑荣集团的搬迁改造前期工作。电缆厂、锅炉厂、轴承厂等企业要按期完成易地搬迁，尽快投产。

　　二是加快农村经济结构调整，大力发展都市农业和生态农业。认真贯彻中央农村工作会议精神，坚持党在农村的基本政策，进一步解决好农业、农村和农民问题。第一，优化农业和农村经济结构，培育农村经济新的增长点，用工业的理念经营农业。加大种植业调整力度，大力发展农业产业化经营。以提高农业竞争力为核心，促进农产品由数量型、低附加值型向质量型、高附加值型转变，促进农业发展、农民增收和农村稳定。认真贯彻退耕还林政策，完成退耕还林18.4万亩。搞好黄河沿线防风治沙生态林建设，争取5年完成40万亩的计划。抓好“食品放心工程”，加快建立健全统一规范的农产品质量标准体系、检验检测体系，抓紧制订农产品生产技术标准，逐步推行农业标准化生产。以实施“安全农产品行动计划”为载体，认真解决蔬菜、鱼、肉以及水果、乳制品的销售安全；严格监控农药的销售和使用；建设一批农业标准化生产示范基地。实行农产品准入制度，严格控制批发市场和市区农贸市场的农产品质量，从根本上解决农产品质量安全和“餐桌污染”问题。第二，统筹城乡经济社会发展，发挥城市对农村的带动作用。各级政府在制定国民经济发展计划、确定财力分配格局时，要把解决好“三农”问题放在优先位置，使城市和农村相互促进、协调发展。加强农村基础设施和生态建设，提高农业的综合生产能力和可持续发展能力。要按照“公平对待、合理引导、完善管理、搞好服务”的方针，为农民进城务工增加收入创造有利条件。第三，推进乡镇企业完成企业产权制度改革，加快建立企业法人治理结构。把结构的战略性调整和技术创新、机制创新和产品的升级换代有机结合起来，高起点跨越式发展高新技术产业，积极培植龙头企业，走贸工农一体化发展道路。积极引导乡镇企业按照严格、科学、高效的原则做好基础管理工作，提高竞争力。第四，以有条件的建制镇为基础，突出重点镇建设，加快城镇化进程。积极稳妥推进城镇化，不刮风，不相互攀比，不盲目扩大建设规模，不搞低水平重复建设。引导乡镇企业、各类专业市场向小城镇集中布局，形成产业支撑，带动和繁荣周边农村经济。

　　三是大力发展现代服务业，完善城市功能。第一，加大改革改制力度，尽快改变国有大型零售商业企业资产结构不合理的状况，继续推进产权多元化，盘活资产，搞活经营。大力优化商业投资环境，认真做好入郑大型商业建设项目的协调服务。第二，进一步治理整顿批发市场。按照全面检查、突出重点、严格监管、完善制度、标本兼治的要求，深入开展各类专项整治工作，严厉打击制假售假等违法犯罪行为。第三，切实做好住房货币化分配工作，降低购房门坎，搞活住房二级市场，调动居民购买住房的积极性。第四，要大力发展旅游业。搞好少林景区拆迁整治，全面推进景点景区建设，拓展旅游市场空间，增强旅游市场竞争力。做好嵩山国家地质公园的揭碑开园和世界文化遗产、世界地质公园的申报工作。第五，积极推进连锁经营、物流配送、电子商务等现代服务业的发展，加快发展现代物流业。大力发展会展经济，高标准建设郑州国际会展中心，搞好全国性、区域性、专业性会展的协调服务工作。

　　四是加快发展非公有制经济，支持个体私营企业发展。进一步采取有效措施，为非公有制企业参与市场竞争创造良好的外部环境。鼓励和引导个体私营企业承包、租赁、收购、兼并国有、集体或其他企业。加快建立面向个体私营企业的社会化服务体系，从政策咨询、资金融通、人才开发、技术交流与创新、信息共享、市场准入等领域支持个体私营企业的发展。

　　(二)加大改革力度，扩大对外开放

　　改革开放是经济发展和社会进步的动力。今年我市的改革开放任务很重，必须加快推进农村、企业、金融和政府机构等重点改革，扩大对外开放，为发展提供强大动力。

　　一是进一步深化各项改革，实现重点领域新突破。巩固和深化农村税费改革成果，确保农民负担不反弹。继续深化粮食流通体制改革，加快粮食购销市场化步伐。积极探索试行将粮食风险金直接补贴给农民的政策，保护农民利益和种粮积极性。

　　推进国有资产管理体制改革。按照中央统一部署，建立国有资产管理机构，探索有效的国有资产经营体制和管理方式，实现国有资产保值增值。深化国有企业改革，大力推进企业的体制、技术和管理创新。按照建立现代企业制度的要求，对国有大中型企业因厂制宜，年内完成规范的公司制或股份制改革，完善法人治理结构。对中原制药厂等长期亏损、资不抵债、扭亏无望的企业，坚决依法实施破产。

　　深化投融资体制改革，吸引更多的社会投资参与经济建设。按照市场经济体制的要求，逐步建立起投资主体自主决策、银行独立审贷、融资方式多样、中介服务规范、政府宏观调控有力的新型投融资体制。树立经营城市的理念，打破政府包建统管的局面，打破部门和行业垄断，吸引社会投资参与交通、水利和城市基础设施建设和经营，解决城市建设资金不足问题。对于教育、文化、卫生、旅游、体育等社会事业，也要建立合理的价格、税费机制，引导民间资本进入。加大对我市改制辅导企业的支持力度，推进成熟企业加快上市步伐，努力扩大股票融资规模。充分发挥金融对经济发展的支持作用。优化信用环境，维护金融债权，防范和化解金融风险。努力扩大信贷消费规模，培育住房、汽车、教育等消费热点。深化农村信用社改革，充分发挥其对农村经济发展的支持作用。

　　深化行政管理体制改革。转变政府职能，改进管理方式，提高行政效率，建立行为规范、运转协调、公正透明、廉洁高效的行政管理体制。继续推进政府机构改革，实现机构和编制的法定化。按照政事分开原则，改革事业单位管理体制。深化行政审批制度改革，依法规范行政审批行为，加强对已取消审批项目的后续监管工作，防止反弹或者变相增加新的审批项目。继续坚持和完善行之有效的集中审批形式和服务方式，提高服务质量和效率。深化人事制度改革，加强公务员队伍管理，实行事业单位全员聘任制，完善人才市场体系建设。

　　二是努力扩大外商投资领域，提高利用外资质量和水平。先，要切实为现有外商投资项目和企业搞好服务，对重点外商和重点项目开辟绿色通道，实现以商招商。努力扩大外商投资领域，积极推进农业、商业、外贸、旅游等领域的对外合作。创新招商方式，在交通、电力、城市公用设施、制造业等领域探索运用项目融资、经营权转让、企业购并、上市公司国有股出让等方式鼓励外商投资。把吸引国际大财团、大企业集团，特别是世界500强投资作为招商引资的重点。其次，要充分发挥两个国家级开发区的政策优势，在利用外资和扩大出口上实现新的突破。加快建设郑州出口加工区，确保2003年封关运行。第三，大力拓展国际市场，巩固和发展传统市场，积极开拓新兴市场。坚持以质取胜，提高商品和服务的竞争力。鼓励有条件的企业“走出去”投资办厂，承包工程。第四，要尽早做好准备，认真办好第8届中国郑州国际少林武术节、郑交会和第18届世界客属恳亲大会；积极开展国际友好城市交流活动，进一步扩大对外友好往来，提高郑州的知名度。

　　三是整顿和规范市场经济秩序，为经济发展创造良好环境。严厉打击制售假冒伪劣商品，特别是制售假劣食品、药品、医疗器械、农资等违法犯罪活动。加强知识产权保护。继续整顿和规范建筑市场、文化市场。加快建立现代社会信用体系，建立企业、中介机构和个人信用档案，逐步实施企业信用公示制，形成“诚信为本、操守为重”的良好风尚。深入贯彻实施《安全生产法》，按照“四不放过”的原则，严肃事故调查处理，加大行政责任追究力度。继续在全市开展以煤矿、消防、交通为重点的安全生产专项整治，逐步建立长效机制。继续坚持“谁主管、谁负责”的原则，全面落实安全生产责任制，最大限度地遏制重特大事故的发生。

　　(三)千方百计扩大就业，健全社会保障体系

　　认真贯彻落实全国再就业工作会议精神，强力实施“一年6万就业计划”，千方百计促进就业和再就业。坚持“劳动者自主择业、市场调节就业、政府促进就业”的方针，着力改善就业环境，坚持大力发展服务业、中小企业和非公有制经济，积极发展劳动密集型产业，提倡灵活多样的就业方式，广辟就业门路，努力扩大就业。加大岗前、在岗和转业(转岗)培训力度，提高下岗失业人员就业和再就业能力。国有企业改革要坚持减员增效与促进再就业相结合，下岗分流与社会承受能力相适应，鼓励国有大中型企业通过主辅分离和辅业改制，分流安置本企业富余人员。抓好区、街、社区劳动保障服务组织建设和再就业培训基地建设，开通“再就业绿色通道”。由于高校扩招，今年我市毕业生将达到2万人，要高度重视和加强对这部分人的就业指导和服务。

　　做好社会保障工作，培育和增强困难群体的消费能力。继续加大社会保险基金的征缴和清理企业欠费工作力度，依法扩大城镇社会保险覆盖面，强化社会保险费支出管理。稳步扩大医疗保险覆盖范围，实施城镇职工生育保险，进一步完善失业保险制度，做好“两个确保”和“三条保障线”的衔接工作。从今年起，国有企业下岗职工不再进入再就业中心，直接进入失业保险。规范城市“低保”工作，切实做到应保尽保，逐步实现低保金的社会化发放。建立和完善对低收入者的救助制度，采取对其子女在义务教育阶段减免学杂费、对双困家庭提供廉租住房及设立低保定点医院等措施，解决他们的实际困难。

　　做好增收减负工作，增强农村居民的消费能力。全面实行涉农税费公示制、贫困地区中小学义务教育一费制、报刊费用限额制和农民负担案件追究制，坚决制止对农民的乱收费。提高种植业收益，大力发展二、三产业，千方百计增加农民收入。引导和支持农民外出务工，取消各种歧视性政策和不合理收费，做好务工服务，依法查处拖欠务工人员工资行为，保证务工农民合法权益。大力推进扶贫开发，确保所有困难群众有饭吃、有衣穿、有房住，不让一个学生因家庭生活困难而失学。

　　(四)加快郑东新区建设，规划改造老城区

　　郑东新区是省市重点建设工程，宏伟蓝图已经绘就，要严格按规划强力推进实施，力争成为全省开放开发的亮点。要抓紧完成各专项规划设计和土地报批，做好国有土地使用权的出让，把项目用地落到实处。加强基础设施建设和河道整治，形成道路骨架和良好的开发环境。中央商务区内，省艺术中心、市广电中心等至少15栋高层建筑要动工建设，房地产开发力争达到150万平方米，学校、医院、商业、文化娱乐网点建设布局合理，初步展示新城区形象。

　　老城区要按照8个专项规划进行改造，着力营造良好的人居环境。年内要重点搞好“两环、三路、两河、两小”工程。“两环”即打通二环路，拓宽改造桐柏路，建设农业路铁路立交；配合省加快建设环城高速公路。“三路”即加紧建设国道220郑州东出口、国道310郑州西出口，年内具备通车条件；开工建设郑州东四环快速路，争取年内完成50%的工程量。“两河”即完成东风渠、熊耳河城区段的综合整治。“两小”即在老城区规划改造50条小街巷、30个小游园。同时，要完成郑少高速公路主体工程和附属物工程建设，年底具备通车条件；加快京珠、少洛高速郑州段的建设。要抓紧完成市区近期建设规划，加强规划监督管理。完成107国道郑州市区段整治。抓好货栈街改造工程、大学校区配套工程等市政工程项目。按照规划切实搞好二七广场治理。加快邙山输水干渠改造工程进度，按期完工。抓好封停、收购自备井工作，加强地下水资源保护。做好兴隆铺热电厂扩建和郑东新区热电厂的前期筹备。搞好西气东输郑州配套管网工程。开工建设五龙口污水处理厂和无害化垃圾处理场。

　　搞好绿化和环境治理，改善生态环境。全力抓好退耕还林工程及平原绿化工程，搞好防护林建设和风沙源治理。加快森林公园、世纪游乐园建设，规划西流湖公园。市区绿化工作除大型绿化重点建设外，要动员全社会见缝插绿，实施立体绿化，增加绿地面积。搞好出入市口的绿化、美化。力争3年左右创建国家园林城市。继续巩固工业污染源达标成果，加强污染防治设施现场监管，遏止污染反弹。以关停不符合环境要求的小造纸为突破口，对全市五小企业进行摸排整顿，解决结构性污染问题。以减少燃煤为主，通过拆除锅炉等燃煤设施，逐步增加天然气等清洁燃料比重，加快城市能源结构调整步伐。继续加大机动车污染防治力度，大力推广车用乙醇汽油。加强供水水源地的水质监测，实施饮用水日报制度，保证市民用水安全。加强小城镇环境保护，开展优美小城镇创建工作，防止污染由城市向农村转移。

　　进一步巩固和完善“两级政府、三级管理、四级网络”城市管理体制，完善城区“四个一”功能，充分发挥区、街在城市管理工作中的主体作用。围绕创建国家卫生城市和全国文明城市，突出“净、绿、亮、美”，做好各项管理工作。积极应用现代信息技术，提高城市基础设施的运行效率和综合效能。继续实施畅通工程，重点整治非机动车和和行人违章，在全市推行大中小学生文明交通卡制度。保证对环卫事业的投入，稳步推进城市维护和市政设施管理社会化、市场化改革，降低城市运作成本。加大环境卫生检查力度，巩固提高道路保洁质量。继续实施亮化工程，加强路灯和夜景照明灯饰的维护管理，保证亮化效果。集中整治城市主要道路景观，拆除违章建筑，清理“烂尾楼”，美化街道建筑立面，整治广告、店牌，提高文化品位。进一步完善城市管理行政执法体制，加强城市管理执法队伍建设，提高依法管理水平，力争市容市貌再上新台阶。

　　(五)做好财政金融工作，支持扩大内需

　　扩大内需是促进经济发展的长期战略方针，要在继续立足于加强基础设施建设，扩大投资需求，增加城乡居民收入，扩大消费需求的同时，认真做好财政金融工作，积极支持扩大内需。

　　依法加强税收征管，重点抓好契税、个人所得税、房产税等财政增收潜力较大的税种，加快建立个人所得税重点纳税人监控体系，提高征管效率，确保收入稳定增长。加大代征代扣力度，建立财源监控网络，加强对重点行业、重点税源的监控。切实加强对非税收入的管理，规范归集财政性资金。强化企业财务管理和会计核算的监督检查，大力清理欠税，严厉打击偷税、逃税、骗税、抗税等违法犯罪行为，确保财政收入与国内生产总值同步增长。

　　坚决反对脱离实际、大手大脚、铺张浪费，严禁新开工建设脱离实际的各种“形象工程”、“政绩工程”。行政机关全面实行“部门预算”。要继续调整财政支出结构，大力压缩一般性支出，退出一般性竞争领域的投资，加大对教育、社会保障、基础设施建设等公共需要的保障力度。各级财政预算安排要确保工资发放。落实专项社会保障补助资金，加大再就业资金投入。进一步强化审计监督工作，深化“收支两条线”管理，行政事业单位的经营性收费要纳入财政管理；完善银行代收制度，实现收缴分离，收支脱钩。

　　认真贯彻执行积极的财政政策和稳健的货币政策，充分发挥金融对扩大内需的支持作用。积极向银行推荐有效益的项目，协调银行落实贷款承诺，支持重点企业和基础设施建设。建立和完善贷款担保机制，帮助中小企业和个体私营企业解决贷款难问题。发展消费信贷，增强居民购房、买车等方面的消费能力。继续支持国内乃至境外各类商业银行、证券公司、保险公司在我市设立分支机构。完善金融监管机制，整顿规范金融秩序，防范化解金融风险。

　　(六)进一步调整教育结构，大力发展各项社会事业

　　积极发展地方高等教育，加快郑州师专新校区、中州大学北校区建设，全力支持郑州大学、信息工程大学二期工程建设和省属高等院校向郑东新区的搬迁。加大农村中小学布局调整力度。全市撤并小学423所，改扩建176所；撤并中学22所，改扩建97所。加快高中阶段教育发展步伐，改建、新建4所优质高中。加强学校体育场馆建设。规范学龄前儿童教育，加强幼儿园管理。引导、扶持社会力量办学。加强职业教育和培训，发展继续教育，构建终身育体系。改革和完善教育人事制度，加强对教师的培养和培训，提高教师的师德和业务水平。

　　加快科技创新体系建设，推进产、学、研结合，不断提高技术创新能力，力争科技进步对国民经济增长的贡献率达到48.3%。实施“2662”工业技术创新计划和“5212”农业技术创新计划。实施制造业信息化工程，改造、提升传统制造业。开展科技进社区活动，带动社区两个文明建设。继续深化科技体制改革，完善科技服务体系，促进科技成果向现实生产力转化。

　　积极推进文化创新，大力发展文化产业。弘扬主旋律，提倡多样化。狠抓艺术精品生产，进一步繁荣文艺创作。继续开展“绿色周末”、广场文化等活动。大力加强基层文化建设，努力探索社区文化工作的新途径和新方法，充分发挥文化工作在提高人民群众素质和城市文明程度上的重要作用。一手抓繁荣，一手抓管理。加强文化基础设施建设，改革完善各类公益设施及场所的管理体制。积极推进卫生体育事业的改革和发展，开展全民健身运动，提高全民健康水平。逐步建立适应新形势要求的卫生服务体系和医疗保健体系，着力改善农村医疗卫生状况，提高城乡居民的医疗保健水平。继续深化计划生育工作综合改革，强化依法管理，提高服务水平，确保人口目标的完成。

　　(七)加强精神文明和民主法制建设，改进政府工作作风

　　深入开展“三个代表”重要思想宣传教育，引导人们树立中国特色社会主义共同理想，树立正确的世界观、人生观和价值观。认真贯彻《公民道德建设实施纲要》，继续开展道德规范进万家活动，广泛进行社会公德、职业道德和家庭美德教育，不断提高人们的思想道德素质。大力开展以创建全国文明城市为重点的精神文明创建活动，继续抓好创建文明社区、文明村镇、文明行业、文明景区工作，进一步提高城乡文明程度。认真组织科技、文化、卫生三下乡活动。崇尚科学，反对迷信，倡导科学文明健康的生活方式。深化全民国防教育，加强民兵预备役建设，争创全国双拥模范城“四连冠”，进一步增强军政、军民团结。

　　坚持依法行政。各级政府要更加自觉地接受人大及其常委会的监督，认真执行人大及其常委会的决定、决议，主动报告工作；加强与人民政协的协商，接受人民政协的民主监督。认真办理人大代表议案、建议和政协委员提案，不断提高办理质量。积极支持工会、共青团、妇联等人民团体开展工作，充分发挥其桥梁纽带作用。重视对台、侨务、民族和宗教工作。认真听取社会各界的合理化建议，发挥各类专家、学者的决策咨询作用，推进决策的科学化、民主化。全面推行依法行政责任制和政务公开，规范行政执法行为。加强执法监督，强化执法人员资格管理，完善行政处罚听证制度，提高依法行政水平。

　　搞好社会治安综合治理，确保社会稳定。按照“分级负责、归口管理”的原则，认真落实各级行政领导保一方平安责任制，努力维护社会稳定。坚持防范在前，加强社会面防控，消除治安隐患。积极推进社区警务建设，整合治安力量，构建群防网络。深入开展严打整治斗争，严厉打击各种刑事犯罪和黑恶势力，坚持扫除黄赌毒等社会丑恶现象，严厉打击、严密防范“法轮功”邪教组织活动，维护社会治安形势稳定。妥善处理人民内部矛盾，保持安定团结的局面。严格控制越级集体上访的发生。

　　反腐败工作是一项长期的、艰巨的任务。各级政府主要领导同志要从关系党和国家生死存亡的政治高度充分认识反腐败斗争的重要性，把党风廉政建设和反腐败工作摆上重要议事日程，切实加强组织领导。按照“谁主管、谁负责”的原则，结合今年反腐败工作重点，将各项任务落实到单位，落实到基层，使之成为广大公务员的自觉行动。各级政府干部要牢记“两个务必”，实践“三个代表”，转变工作作风，继承发扬艰苦奋斗的优良传统，带领群众奔小康。

　　进一步改进管理方式，提高管理水平。更多地运用经济和法律手段管理经济和社会事务，减少对市场主体的直接干预。继续推行和完善“一个窗口对外”、“一条龙服务”等各项服务，提高行政效率。要完善重大决策的规则和程序，推进决策的科学化、民主化。继续抓好政府办公自动化，大力推进电子政务建设，保证社会对政府工作的知情权，提高透明度和办事效率，方便群众监督。继续深入开展“为纳税人服务”和“社会各界评议政府部门”活动。进一步加强公开办事大厅的建设，建立健全服务公开制、服务承诺制、限时办结制、首问责任制、失职追究制等相关制度，提高行政效率。

　　贴近人民生活，集中力量办好十件实事。紧紧围绕群众关切的热点难点问题，制定并承诺为老百姓办好实事，是近些年来我们行之有效的工作方法。这一做法充分表达了人民的意愿，增强了政府工作的透明度，体现了人民政府为人民的宗旨，符合“三个代表”重要思想的要求，便于督促检查，受到广大群众和社会各界的普遍欢迎。经过充分征求人民代表和各县(市)区、各部门的意见，今年为人民群众再办十件实事：1.拓宽就业渠道，新增6万个就业岗位。2.完成市区50条背街小巷改造，改造市区100所旱厕，完成30处街头游园配套建设。3.开工建设二环路工程，改善100公里县乡公路路况。4.启动住房货币化分配制度，向社会提供30万平方米经济适用房，廉租住房覆盖率达到符合条件“双困”家庭的90%以上。5.市区环境空气质量达到功能区标准；新增燃气用户2.2万户、集中供热面积30万平方米；增加更新公交车150台，改造天然气公交车150台。6.进一步调整中小学校布局，解决城区80%中小学校采暖、降温设施；改造农村中小学现有危房3万平方米；市直学校完成“校校通”信息网工程。7.启动农产品市场准入，发布农产品质检日报。8.全市每所乡镇卫生院配备1台B超诊断仪及1台救护车；建成100个乡镇规范化计划免疫预防接种门诊。9.西部山区建1万个集雨节灌水窖，扩大旱保田2万亩。10.建成残疾人康复中心；确保所有盲道畅通使用，大型公共设施改造为无障碍设施。这十件实事要列入各有关部门责任目标，进行督促落实和考核，把实事办好，好事办实。

　　各位代表，我们正站在新的历史起点上，向着更高的目标前进。展望郑州未来，前景无限美好。让我们紧密团结在以胡锦涛同志为总书记的党中央周围，高举邓小平理论伟大旗帜，全面贯彻“三个代表”重要思想，认真落实十六大精神，万众一心，与时俱进，开拓进取，为全面建设小康社会、开创我市社会主义现代化建设的新局面而共同奋斗！
